# Supplementary material for: Productive wetlands restored for carbon sequestration quickly become net CO2 sinks with site-level factors driving uptake variability
Source: PLoS One. 2021 Mar 25;16(3):e0248398. doi: 10.1371/journal.pone.0248398 (PMC7993764; doi:10.1371/journal.pone.0248398)
Supplement: S1 File — (DOCX) [file pone.0248398.s001.docx]

Supplemental information: Productive wetlands restored for carbon sequestration become effective CO_2_ sinks depending on design and management factors

A.C. Valach, K. Kasak, K.S. Hemes, T. L. Anthony, I. Dronova, S. Taddeo, W. L. Silver, D. Szutu, J. Verfaillie, and D. Baldocchi

## **S1 Land cover classification comparison**

To test the impact of image resolution on the relationship of vegetation cover and carbon (C) fluxes, a fine-scale classification was done using very high resolution (0.05 m) radiometrically corrected and rectified aerial images from a single flight collecting LiDAR measurements in September 2018 [1] and compared with the lower resolution land cover classification from the corresponding 2018 growing season. Again, a supervised learning approach using the Support Vector Machine tool in ArcGIS Pro v2.4 was applied to segmented images and, where necessary, objects manually reclassified based on the original high-resolution aerial image. Due to the high resolution of the images, individual vegetation types could be discerned, which were split into live vegetation (predominantly emergent macrophytes with some grasses and individual trees), floating aquatic vegetation (*Azolla*), algae, as well as live, dead, or densely mixed live and dead biomass. Areas of open water with submerged vegetation not visible at the surface were not included. The vegetation types were compared with C fluxes, in which vegetation classes were also grouped into photosynthesising (green) vegetation and total vegetation. The land cover maps were created using ArcGIS® software by Esri (Copyright © 2019 Esri Inc. All rights reserved).

The coarse and fine-scale classifications of total vegetation cover (Figure S1) compared well with each other for all sites (slope = 0.92, R^2^ = 0.98, p <0.01) and linear regressions with monthly and annual NEE and GEP were somewhat improved (Table S1). Only East Pond and Sherman Wetland differed by 7% and 19%, respectively.

**
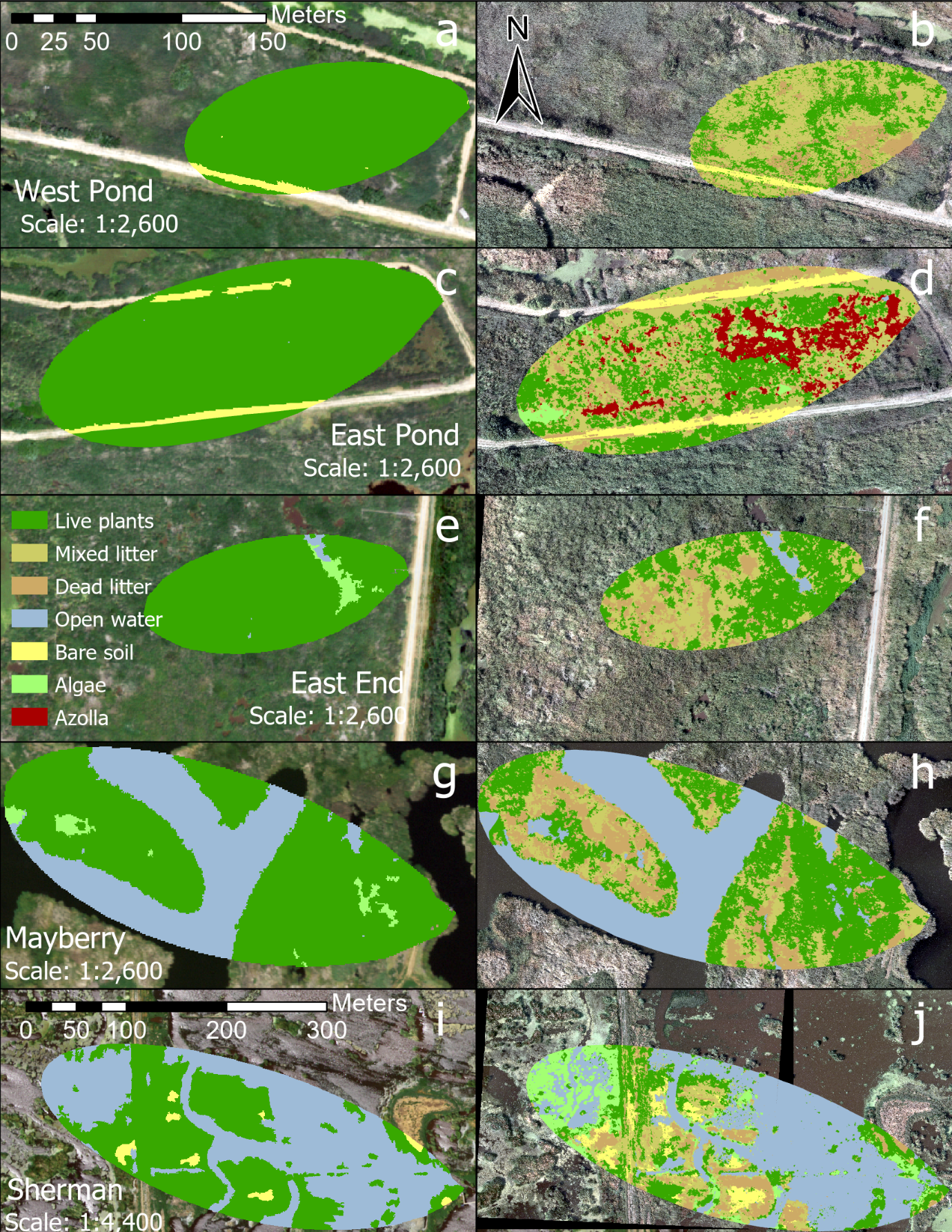
**

**Figure S1. Land cover classifications for different image resolutions.** Coarse (left) and fine-scale (right) classifications of water, bare ground, and various vegetation types (live vegetation, dead litter, mixed live and dead vegetation, *Azolla*, and algae) in the flux footprints at West Pond (a, b), East Pond (c, d), East End (e, f), Mayberry (g, h), and Sherman Wetland (i, j) during 2018 scaled to 1:2,600 (a-h) or 1:4,400 (i and j). Data were visualised using ArcGIS Pro v2.4 (ArcGIS® software by Esri Copyright © 2019 Esri Inc.) using open access images from the National Agriculture Imagery Program (left) and our LiDAR flight (right) in 2018.

**S1 Table. Vegetation cover classification relationships with corresponding cumulative monthly, annual, and mean interannual net ecosystem exchange (NEE) and gross ecosystem productivity (GEP) fluxes for all site-years with the corresponding classification image.**

|  | *Total vegetation cover* | | *September 2018 images* | |
| --- | --- | --- | --- | --- |
| *Flux* | *Time scale* | | *Coarse* | *Fine* |
| *NEE (gC CO_2_ m^-2^)* | *Monthly sum (m^-1^)* | *R^2^* | 0.52 | 0.66 |
|  |  | *p* | 0.167 | <0.1’ |
|  | *Annual sum (yr^-1^)* | *R^2^* | 0.34 | 0.47 |
|  |  | *p* | 0.31 | 0.20 |
| *GEP (gC CO_2_ m^-2^)* | *Monthly sum (m^-1^)* | *R^2^* | 0.41 | 0.54 |
|  |  | *p* | 0.25 | 0.16 |
| *GEP (gC CO_2_ m^-2^)* | *Annual sum (yr^-1^)* | *R^2^* | 0.38 | 0.50 |
|  |  | *p* | 0.27 | 0.18 |

Two different image resolutions were compared in 2018, which was also used to compare the overall site-means. Significance levels are *** 0.001, ** 0.01, * 0.05, 0.1’.

## **S2 Canopy height measurement comparison**

For the canopy height method comparison data from ground surveys, aerodynamic height measurements and LiDAR data from August and September 2018 were used.

LiDAR measurements (Titan Lidar, Teledyne Optech, Toronto, Canada) were used to produce digital elevation and surface models, which are 3D model representations of the underlying terrain (elevation) and the top surfaces of aboveground objects (surface), respectively. The difference between these two models were used to estimate vegetation height, structure, and litter layers at the wetland sites. This resulted in a height difference of above-ground objects, which means areas of open water without surface vegetation did not influence the average canopy height.

Parametric ANOVAs with Tukey’s Honest Significant Difference tests and non-parametric Kruskal-Wallis tests with Dunn’s multiple comparisons (Z-statistic) were used for categorical comparisons (with factor “method”) of vegetation height measurement methods between sites due to the unequal sample sizes [2]. All canopy height frequency distributions followed a normal distribution, except sites with floating vegetation or larger areas of dry land covered with short-statured plants, which were skewed, such as East Pond and Sherman Wetland (skew of 2.8 and 3.5 respectively).

To distinguish canopy heterogeneities within the vegetation patches, the vegetation height above the water level and canopy structure were analysed using three methods (Table S1); 1) LiDAR measurements provided elevation points used to estimate canopy height and structure (Figures S2), 2) the aerodynamic canopy height was calculated from eddy covariance turbulence measurements, and 3) ground transects within the flux footprint were used as a direct *in situ* comparison.

The comparison of methods to estimate canopy heights showed that the aerodynamic canopy heights compared well with ground transects indicating this to be an effective and readily-available approach to estimate canopy heights from heterogeneous marsh vegetation. Previously, this method has been successfully tested over agricultural crops, grasslands, and at forest sites [3,4]. Due to the high temporal frequency of this method, it can be used to show vegetation establishment over time.

The dead litter layer within the canopy was the source of the underestimation of LiDAR canopy heights, especially at sites with tall dense canopies as the laser pulses missed the tips of the emergent macrophytes because of the small nadir view of leaf surface area of these erectophyle plants. The LiDAR effectively measured the litter heights, whereby the LiDAR canopy heights were comparable to, but still lower than, the ground measured litter layer (Table S2). Other studies using LiDAR to measure *Typha* spp. canopy heights showed good agreement with ground measurements, however, plants were considerably shorter (0.7-1.5 m) and measurements were taken in October [5]. Aerodynamic canopy height measurements lack granular information on the spatial distribution of vegetation height, which can also vary within vegetation types. Particularly at sites with open water channels, the emergent macrophytes tended to be taller along the edges than within the vegetation patch [6,7], which was visible to some degree in the LiDAR images (Figure S2). All three methods have distinct benefits and limitations in terms of practicality, costs, and spatial and temporal representativeness, yet the aerodynamic method provided a good compromise capturing an accurate median canopy height within the footprint with a high temporal resolution and minimal effort and costs. It also has the capacity to monitor seasonal blow-downs in vegetation that help bury the dead material into the water column, forming peat and reducing light attenuation.

**
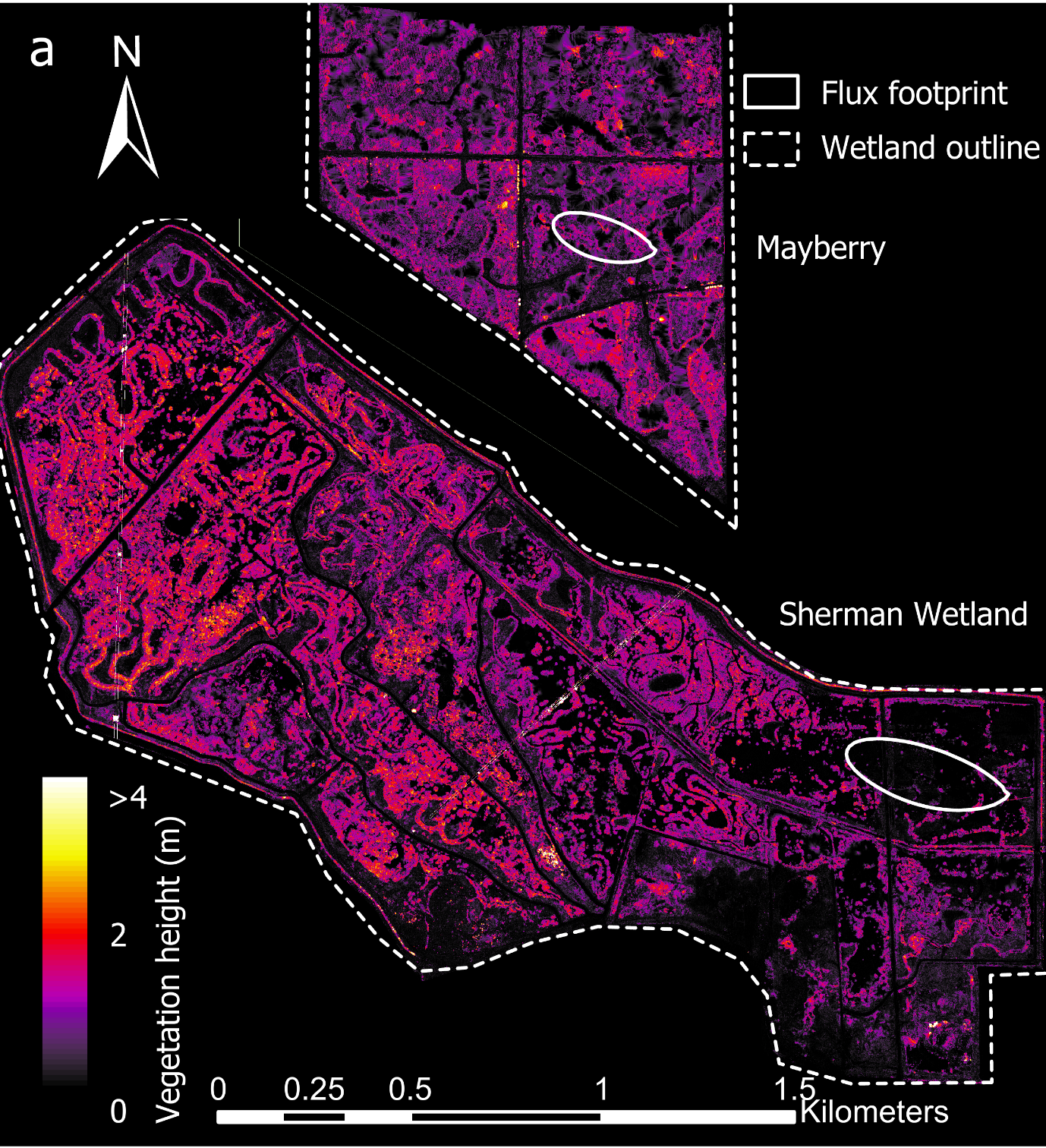
**

**
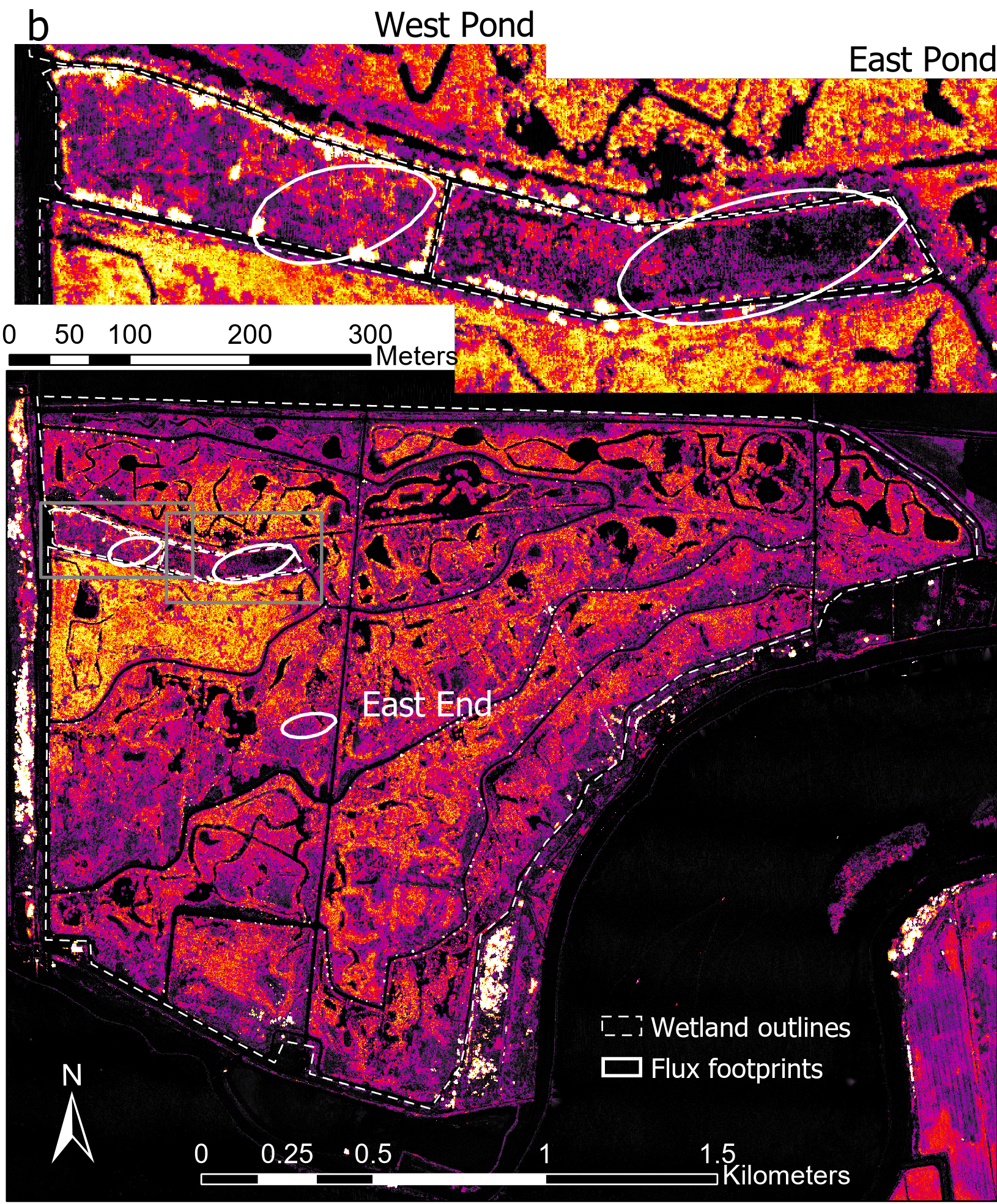
****Figure S2.** **Maps of calculated vegetation height (m) using the LiDAR data from September 2018** **from a) Sherman Island with Mayberry and Sherman Wetlands and b) Twitchell Island, which includes East End, with East Pond and West Pond** shown as insets plus the neighbouring wetland where flux footprints extend beyond the boundaries. Colour bars show the enhanced gradient for heights between 0 (black) and >4 m (white). White outlines show the wetland extents (dashed line), as well as the 85 % flux footprints (solid line).

**S2 Table. Comparison of median and interquartile range (IQR) canopy heights measured by ground transects, LiDAR, and calculated aerodynamic canopy height from turbulence statistics at all wetland sites in August - September 2018.**

| ***Site*** |  | ***Median (IQR) canopy height (m)*** | | |
| --- | --- | --- | --- | --- |
|  | *Ground transect* | *Ground litter* | *LiDAR* | *Aerodynamic** |
| *East End* | 3.00 (2.81 - 3.40) | 1.30 (1.10-1.70) | 1.14 (0.73 - 1.57) | 2.77 (2.66 - 2.91) |
| *East Pond* | 1.42 (1.30 – 1.82) | 0.75 (0.55 – 1.02) | 0.39 (0.06 - 0.99) | 2.22 (2.09 – 2.40) |
| *West Pond* | 3.80 (3.30 – 4.00) | 1.70 (1.40 – 2.00) | 1.15 (0.62 – 1.72) | 3.05 (2.94 – 3.28) |
| *Mayberry* | 1.85 (1.40 – 2.30) | 1.22 (1.01 – 1.39) | 0.65 (0.29 – 1.04) | 1.85 (1.75 – 1.96) |
| *Sherman Wetland* | *NA* | *NA* | 0.04 (0.02 – 0.12) | 0.18 (0.16 – 0.26) |

*NA* No ground transect data were available for Sherman Wetland.

*Numbers are integrated daily medians.

The height difference model in Figure S2 based on LiDAR-derived canopy heights clearly indicates that canopies were highly heterogeneous within each wetland with very flat areas (black), such as open water and bare ground (including roads and levees) and short-statured pastures adjacent to the wetlands compared to areas with tall wetland vegetation (colour scale with darker colours showing shorter and lighter colours taller structures). The LIDAR measurements also allowed a comparison of the canopy representativeness between the flux footprint and the whole site, which showed that canopy heights in Sherman Wetland were distinctly different within the footprint compared to the rest of the site (mean footprint canopy height of 0.009 ± 0.24 m with a site mean canopy height of 0.558 ± 0.83 m).

Between-site differences in canopy heights (Table S2) were statistically significant for all methods (absolute Z-statistics between 29.25 to 132.54, p <0.001) with East End and West Pond being the most similar (Z-statistic = 1.95, p = 0.051). Canopy height measurements varied between methods at all sites (absolute Z-statistics between 2.73 to 132.17, p <0.01) bar East End and West Pond using LiDAR (Z-statistic = 1.90, p = 0.057) and ground measurements (Z-statistic = -0.80, p = 0.427), as well as East Pond and Mayberry for ground measurements only (Z-statistic = -1.29, p = 0.235).

Canopy heights measured by LiDAR were on average significantly lower compared to ground measurements by 1 - 2 m representing a three-fold reduction (Z-statistics between 4.82 and 43.11, p <0.001). LiDAR-derived heights were also lower than aerodynamic canopy heights (Z-statistics between 9.37 and 12.74 , p <0.001) with a median difference of 1.41 m (ranging between 0.15 m at Sherman Wetland and 1.75 m at West Pond). The median aerodynamic canopy heights compared well with ground transects and were not statistically different.

## **S3 Soil sampling**

### **S3.1 Soil sample processing**

Samples were taken in August 2018 as a one-time characterisation of the surface soil C, N, and P content from 0-15 cm depth at each site. Ten samples were collected using sediment cores at each site across two transects within the flux footprint, with each sampling location at least 3 m apart. All soil samples were immediately bagged, stored at 4 °C, and air-dried at room temperature in the laboratory. Air-dried samples were sieved to 2 mm and all visible roots were removed. These samples were then ground to a fine powder and analysed in duplicate for total C and N using an elemental analyser (CE Elantech, Lakewood, NJ, USA). Soil organic (P_o_) and inorganic (P_i_) P pools were determined by sequential extraction with 0.5 M sodium bicarbonate (NaHCO_3_, 1 g organic dry weight fresh soil in 45 mL solution) and 0.1 M sodium hydroxide solution (NaOH, 45 mL solution) [8]. Total P in both extracts (NaHCO_3_-P_t_ and NaOH-P_t_) was determined by measuring PO_4_ according to the standard colorimetric method [9] after autoclaving extracted solutions with ammonium persulfate (NH_4_)_2_S_2_O_8_. Inorganic P was also determined in the NaOH extract (NaOH-P_i_) [9] after acidifying and centrifuging the extractant. Organic P in the NaOH extract (NaOH-P_o_) was estimated by subtracting inorganic P from total P in the extracts.

### **S3.2** **Soil chemical properties**

All sites have high concentrations of C and N in surface soils (Figure S3). Sherman Wetland values were lower (with mean ± SD of 4.7 ± 0.7% C, 0.36 ± 0.06% N) than the other sites (11.3 ± 1.9% C to 20.6 ± 4.5% C, 0.68 ± 0.07% N to 1.29 ± 0.28% N). Average C:N ratios were 15.5 ± 0.9, 15.9 ± 1.0, 16.7 ± 1.5, 15.7 ± 0.8, and 13.1 ± 0.2 for West Pond, East Pond, East End, Mayberry, and Sherman Wetland, respectively. These values were consistent with the USDA soil survey map indicating Rindge muck Histosols at the tower sites [10]. East End and Sherman Wetland also had larger extents of Gazwell and Scribner Mollisols.


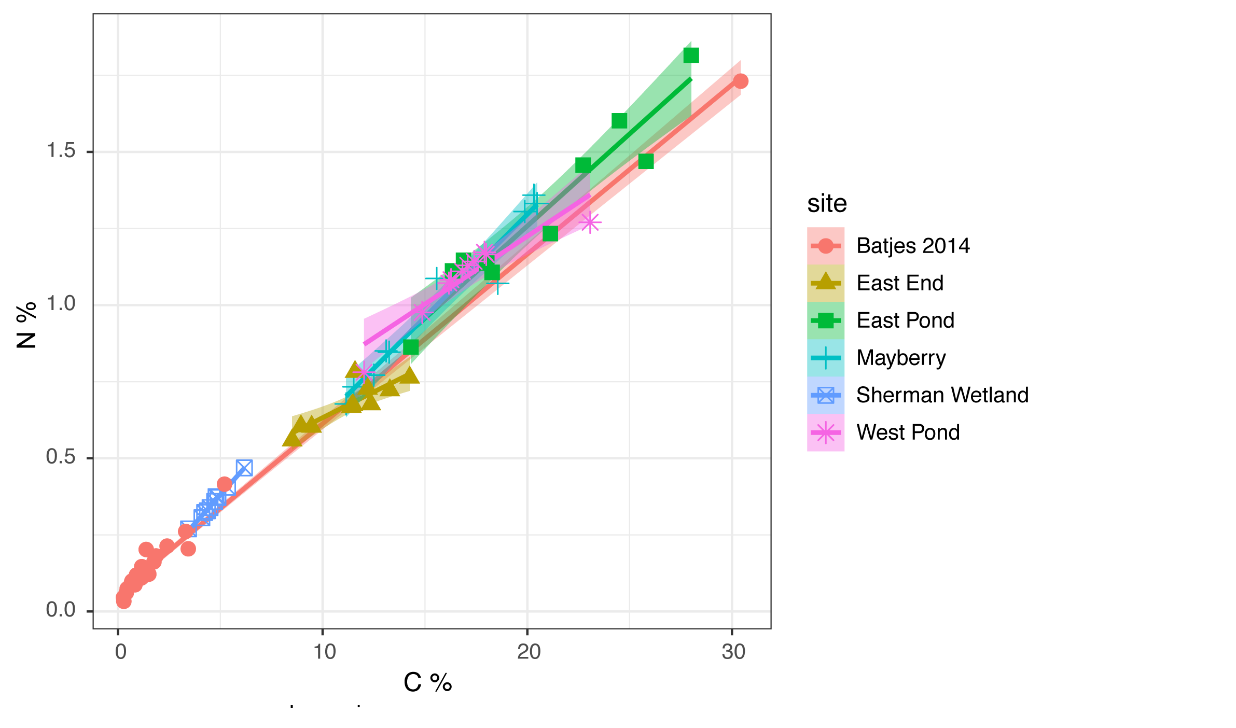


**Figure S3.** **Percent soil C and N content in the Delta wetlands.** Values were determined from topsoil (0-15 cm) samples compared with 26 major soil units of the world [11].

The percent of C and N were positively correlated (R^2^ = 0.97, p <0.001) and fit on the existing regression line [11], which consisted of 26 major soils around the world (Figure S3). East Pond, West Pond, and Mayberry were in the upper range, while East End and Sherman Wetland were the lowest of our wetlands, but still higher than most other soil types.

Concentrations of NaOH P_i,_ generally associated with amorphous and crystalline Fe and Al minerals, was highest at East End (Figure S4a). This site is partially underlain by Fe-rich alluvium deposited by historic runoff from the northern Sierra Nevada range [12]. Both East End and West Pond NaOH-P_i_ concentrations were higher than Mayberry. Concentrations of NaOH-P_o_ was also significantly higher in West Pond compared with Sherman Wetland (Figure S4b). There were no statistically significant differences in NaHCO_3_-extractable P_i_ across the wetlands (Figure S4c). Average NaHCO_3_-P_o_ concentrations were significantly higher in East End compared to both Sherman Wetland and Mayberry (Figure S4d).


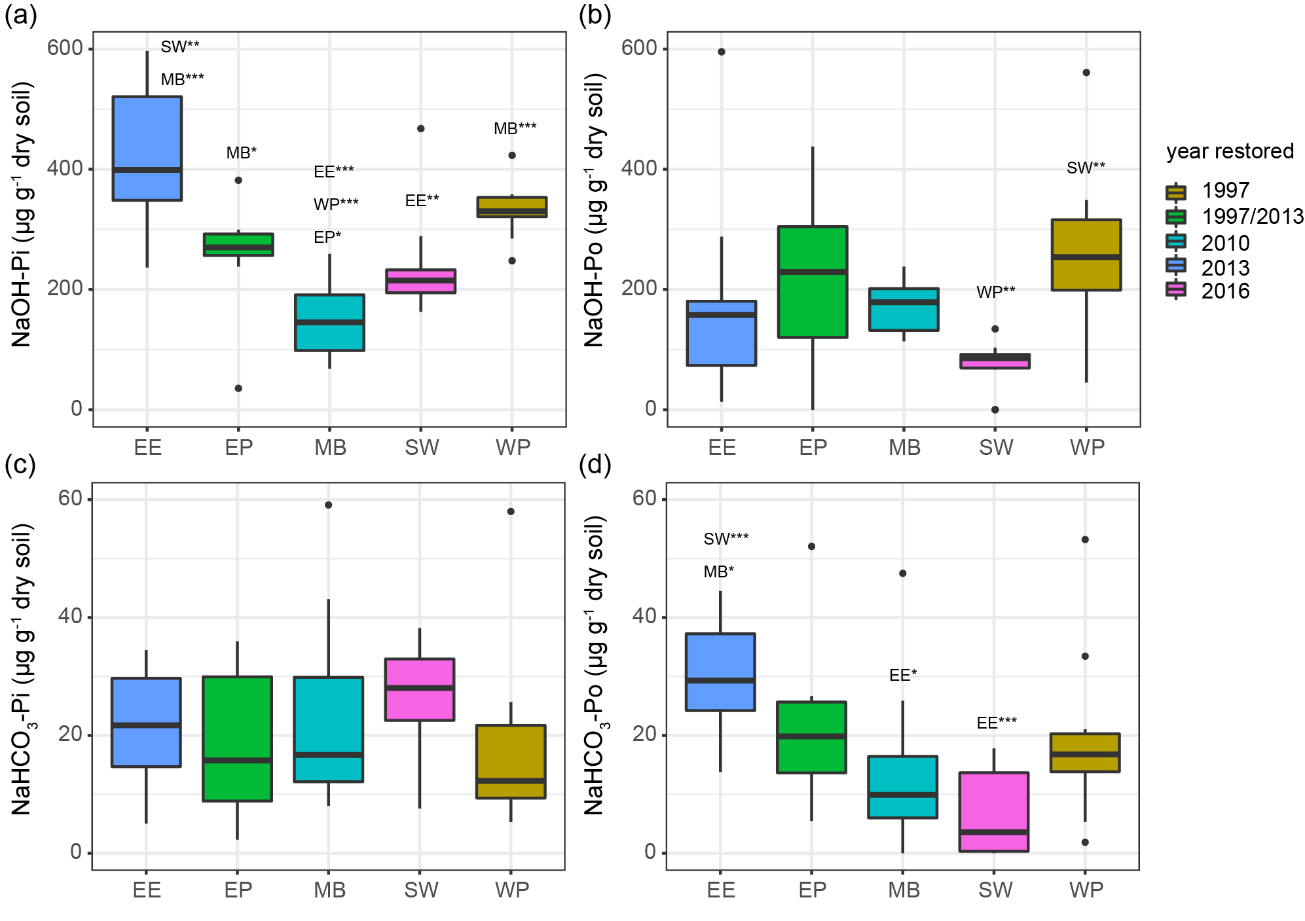


**Figure S4.** **Boxplots of (a) NaOH extractable inorganic P (NaOH-P_i_), (b) NaOH extractable organic P (NaOH-P_o_), (c) NaHCO_3_-extractable inorganic P (NaHCO_3_-P_i_), and (d) NaHCO_3_-extractable organic P (NaHCO_3_-P_o_) across all wetland sites coloured by restoration year.** The bars indicate median values with interquartile ranges. Multiple comparisons were controlled by Tukey’s HSD tests with asterisks denoting statistically significant differences: *** p <0.001, ** p <0.01, * p <0.05. Labels are East End (EE), East Pond (EP), Mayberry (MB), Sherman Wetland (SW), and West Pond (WP).

Generally, mature wetlands showed the lowest NaOH- and NaHCO_3_-extractable P concentrations and higher organic P. Concentrations of NaHCO_3_-P_i_ and NaOH-P_o_ were significantly positively correlated with monthly sums of NEE in August 2018 (R^2^ = 0.82 and R^2^ = 0.84, p <0.05, respectively) across all sites.

### **S3.3** **Discussion of potential soil nutrient impacts**

All sites had high C and N concentrations compared to a global soil database [11], as well as high inorganic and organic forms of phosphorus (P_i_ and P_o,_ Figure S3). Although not measured here, sites may also be rich in inorganic N through continuous groundwater or surface water intrusion of N-rich agricultural run-off. Since the restored marshes are interspersed among highly fertilised agricultural fields [13], the nutrient movement through groundwater seepage and pumping river water may be increasing the nutrient concentrations in the restored wetlands affecting the restoration trajectory [14].

Overall, both NaOH (Figure S4a and b) and NaHCO_3_ (Figure S4c and d) extractable P_i_ and P_o_ concentrations were high across all the wetlands. NaHCO_3_ extractable P is weakly adsorbed on soil particles and therefore generally assumed to be readily available to plants [15], while NaOH extractable P is chemically sorbed to secondary iron (Fe) and aluminium (Al) minerals and is likely to be more stable [16]. Both NaOH pools are associated with Fe and Al minerals, however, NaOH-P_i_ in inorganic form, such as phosphate and NaOH-P_o_, is incorporated in organic matter, which includes fluvic and humic acids. Both NaOH-extractable P_i_ and P_o_ have intermediate lability, which is thought to be available to plants and microbes over longer timescales [17]. Although Sherman Wetland has similar soil configurations as East End, the previous land use management may explain the differences in NaOH P_i_ concentrations. East End was planted with corn and highly fertilised, whereas Sherman Wetland was a degraded pasture (mean ± SD NaOH P_i_ of 599 ± 105.8 and 298 ± 82.6 µg g^-1^ dry soil, respectively). Although more mature, both West Pond and East Pond were also originally planted with corn, as well as being surrounded by highly fertilised corn fields until the restoration of East End. As restored wetlands age, it would be expected that NaOH P_i_ is extensively utilised by both plants and microbes converting it into less available NaOH P_o_ pools. Higher NaHCO_3_-extractable P_i_ in young wetlands, such as Sherman Wetland, may indicate that plant available P was more abundant where vegetation was still establishing. In contrast, the recent rapid growth and litter input at East End may have resulted in higher NaHCO_3_-extractable P_o_ compared to the slower vegetation development at the other two young sites.

Overall, wetland age, as well as historic land use may have contributed to the patterns in extractable P at these sites, but a single sampling campaign was insufficient to draw conclusions of soil P concentrations over longer timeframes.

## **References**

| 1. Hemes K. (2019). Ecosystem Structure in Hetereogeneous Restored Wetlands: Sherman Island, CA. National Center for Airborne Laser Mapping (NCALM). Distributed by OpenTopography, Compiler. Dataset: doi.org/10.5069/G9ZK5DT3 |
| --- |
| 1. Dunn O. Multiple comparisons using rank sums. Technometrics, 1964; 6, 241-252. |
| 1. Chu H, Baldocchi DD, Poindexter C, Abraha M, Desai AR, Bohrer G, et al. Temporal Dynamics of Aerodynamic Canopy Height Derived from Eddy Covariance Momentum Flux Data Across North American Flux Networks. Geophys Res Lett, 2018; 45(17), 9275–9287. https://doi.org/10.1029/2018GL079306 |
| 1. Pennypacker S, Baldocchi D. Seeing the Fields and Forests: Application of Surface-Layer Theory and Flux-Tower Data to Calculating Vegetation Canopy Height. Boundary-Layer Meteorol, 2016; 158, 165-182. |
| 1. Gilmore MS, Wilson EH, Barrett N, Civco DL, Prisloe S, Hurd JD, et al. Integrating multi-temporal spectral and structural information to map wetland vegetation in a lower Connecticut River tidal marsh. Remote Sens Environ, 2008; 112, 4048–4060. doi:10.1016/j.rse.2008.05.020 |
| 1. Bonneville MC, Strachan IB, Humphreys ER, Roulet NT. Net ecosystem CO2 exchange in a temperate cattail marsh in relation to biophysical properties. Agric For Meteorol, 2008; 148(1), 69–81. https://doi.org/10.1016/j.agrformet.2007.09.004 |
| 1. Dronova I, Taddeo S. Canopy Leaf Area Index in Non-Forested Marshes of the California Delta. Wetlands, 2016; 36, 705–716. |
| 1. Tiessen H, Moir JO. Characterization of available P by sequential extraction. In M. R. Carter (Ed.). Soil sampling and methods of analysis, Canadian Society of Soil Science, Boca Raton, FL: Lewis Publishers, 1993; 75-86. |
| 1. Murphy J, Riley JP. A modified single solution method for the determination of phosphate in natural waters, Anal Chim Acta, 1962; 27, 31–36. Doi:10.1016/S0003-2670(00)88444-5 |
| 1. NRCS (Natural Resources Conservation Service), United States Department of Agriculture. Web Soil Survey. Available online at the following link: https://websoilsurvey.sc.egov.usda.gov/. Accessed [10/2/2019]. |
| 1. Batjes NH. Total carbon and nitrogen in the soils of the world. European J Soil Sci, 2014; 65(1), 10-21. doi:10.1111/ejss.12114_2 |
| 1. Graham RC, O'Geen AT. Soil mineralogy trends in California landscapes. Geoderma, 2010; 154(3-4), 418-437. doi:10.1016/j.geoderma.2009.05.018 |
| 1. Almaraz M, Bai E, Wang C, Trousdell J, Conley S, Faloona I, et al. Agriculture is a major source of NOx pollution in California. Sci Adv, 2018; 4(1). doi:ARTN eaao347710.1126/sciadv.aao3477 |
| 1. Kill K, Pärn J, Lust R, Mander Ü, Kasak K. Treatment Efficiency of Diffuse Agricultural Pollution in a Constructed Wetland Impacted by Groundwater Seepage. Water, 2018; 10(11). doi:Artn 160110.3390/W10111601 |
| 1. Hedley MJ, Stewart JWB, Chauhan BS. Changes in Inorganic and Organic Soil-Phosphorus Fractions Induced by Cultivation Practices and by Laboratory Incubations. Soil Sci Soc Am J, 1982; 46(5), 970-976. doi:10.2136/sssaj1982.03615995004600050017x |
| 1. McLaughlin JR, Ryden JC, Syers JK. Development and Evaluation of a Kinetic-Model to Describe Phosphate Sorption by Hydrous Ferric-Oxide Gel. Geoderma, 1977; 18(4), 295-307. doi:10.1016/0016-7061(77)90038-6 |
| 1. Richter DD, Allen HL, Li J, Markewitz D, Raikes J. Bioavailability of slowly cycling soil phosphorus: Major restructuring of soil P fractions over four decades in an aggrading forest. Oecologia, 2006; 150(2), 259–271. https://doi.org/10.1007/s00442-006-0510-4 |
